# Meta-analysis of Germline Whole-exome Sequencing in 1435 Cases of Testicular Germ Cell Tumour to Evaluate Disruptive Mutations Under Dominant, Recessive, and X-linked Inheritance Models

**DOI:** 10.1016/j.euros.2025.01.015

**Published:** 2025-02-13

**Authors:** Zeid Kuzbari, Charlie F. Rowlands, Isaac Wade, Alice Garrett, Chey Loveday, Subin Choi, Beth Torr, Kevin Litchfield, Alison Reid, Robert Huddart, Peter Broderick, Richard S. Houlston, Clare Turnbull

**Affiliations:** a Division of Genetics and Epidemiology, The Institute of Cancer Research, London, UK; b Department of Clinical Genetics, St George’s University Hospitals NHS Foundation Trust, London, UK; c Cancer Research UK Lung Cancer Centre of Excellence, University College London Cancer Institute, London, UK; d Tumour Immunogenomics and Immunosurveillance Laboratory, University College London Cancer Institute, London, UK; e Academic Radiotherapy Unit, The Institute of Cancer Research, London, UK

**Keywords:** Cancer susceptibility genes, Gene association testing, Germ cell tumour, Germline mutations, Meta-analysis, Testicular cancer, Whole-exome sequencing

## Abstract

**Background and objective:**

Testicular germ cell tumour (TGCT) is the most common cancer in young men, and over half of its high estimated heritability is unexplained. Our objective was to identify rare pathogenic germline variation driving TGCT susceptibility.

**Methods:**

This study is a case-control meta-analysis of whole-exome sequencing data from three datasets (Institute of Cancer Research, The Cancer Genome Atlas, and UK Biobank). We retained unrelated male individuals of European ancestry comprising 1435 TGCT cases and 18 284 cancer-free controls. We performed gene-level association testing of protein-truncating variants and nonsynonymous disruptive variants across six candidate gene sets (733 genes) potentially biologically related to TGCT. We then analysed exome wide (19 355 genes) under dominant and recessive models, including X-linked genes.

**Key findings and limitations:**

No individual gene-disease association was identified following multiple testing corrections. However, functional gene-set analyses identified an excess of associations with genes involved in microtubular/ciliary pathways (*p* = 1.69 × 10^–8^). Our study was well powered to detect rare variation of moderate/high effect sizes (odds ratio [OR] ≥5), but power diminished for modest effect sizes (OR <5).

**Conclusions and clinical implications:**

Although this is the largest whole-exome analysis of TGCT to date and first exome-wide examination for recessively acting gene associations, larger studies are required to identify robust associations for individual genes.

**Patient summary:**

We investigated samples from 1435 men with testicular cancer and 18 284 men without cancer to compare the rate of disruptive mutations in 19 355 genes. No evidence of specific genes associated with testicular cancer was discovered, although one gene group showed a strong association. Larger studies are needed to identify individual genes associated with causing testicular cancer.

## Take Home Message

This is the largest whole-exome study of testicular germ cell tumours (TGCTs) comprising 1435 cases. Although one gene group showed a strong gene-disease association, no such individual associations were identified, thus requiring larger studies to identify the genetic basis driving TGCT susceptibility.

## 1 Introduction

Testicular germ cell tumours (TGCTs) are the most common cancer in men aged 15–44 yr, with a one in 200 lifetime risk and >74 000 cases per year worldwide [1], [2]. TGCT has a strong heritable basis (37–49%), which is reflected in the four- to six-fold increased risk in first-degree male relatives of cases.

Genome-wide association studies (GWASs) of TGCTs have been amongst the most productive studies across all cancers, with >44% of the heritability explained by the 78 TGCT-associated loci identified to date [3], [4]. In contrast to this success, despite multiple linkage and case-control studies, attempts to delineate TGCT susceptibility genes acting via rare moderate- to high-risk disease-causing variants have been less productive (Supplementary Table 1) [5], [6].

In segregation analysis of exome-wide sequencing data from 153 multicase TGCT families, we previously found evidence in two pedigrees of segregation with TGCTs for *DNAAF1*. The implication of *DNAAF1*, biallelic mutations that are reported to cause ciliary dyskinesia in humans, as a TGCT susceptibility gene was supported by data from functional studies and animal models [7]. However, our subsequent exome-wide analysis of 919 predominantly unselected TGCT cases and 1609 controls identified no significant associations for *DNAAF1* or any other gene after multiple testing correction [8]. *CHEK2,* a well-established breast cancer susceptibility gene with moderate-risk associations, was also reported for multiple other cancer types [9]. In a case-control analysis of ten DNA repair genes involving 205 TGCT cases, rare pathogenic *CHEK2* variants were reported to confer a four-fold risk of TGCT [10]. In the largest familial TGCT study to date, a recent analysis of 228 familial probands reported candidate association between TGCTs and loss-of-function (LoF) variants in ten genes, and implicated pathways related to cotranslational protein targeting, chromosomal segregation, and sex determination [11]. Despite the advent of next-generation sequencing (NGS) enabling more comprehensive analyses for TGCT-associated genes, as yet there has been little independent validation of the multiple reported associations (Supplementary Table 2). The search for rare variants causative of TGCTs has to date been conducted assuming a dominant model of predisposition. However, the higher relative risk of TGCTs between brothers, than that in parent-son relationships, has long indicated the distinct possibility of recessive and/or X-linked inheritance models.

To address the shortcomings of previous searches, we report the largest case-control TGCT susceptibility gene discovery analysis to date, comprising 1435 TGCT cases and 18 284 controls analysed under dominant and recessive models. This analysis incorporates 667 TGCT cases from previously reported Institute of Cancer Research (ICR) studies, combining them with new data from 768 TGCT cases from UK Biobank (UKB) and The Cancer Genome Atlas (TCGA). Importantly, our power is particularly improved by making use of a new and much larger control dataset from UKB comprising 18 284 cancer-free controls (compared with 1609 in our previous analysis). Combination in a meta-analysis would thus elevate statistical power to empower identification of novel gene associations. Furthermore, by applying frequency weighting, we have included variants spanning a broad spectrum of allelic frequency; we therefore present for the first time an association analysis of common, low-frequency, and rare coding variants.

## 2 Patients and methods

### 2.1 Studies, patients, and data

Our analysis comprised three TGCT case series: (1) 986 previously reported cases recruited at the ICR [7], [8], (2) 150 cases recruited via the TCGA program [12], and (3) 658 UKB cases (see the Supplementary material for details of recruitment studies) [13]. For controls, we used exome data from 18 400 randomly selected male individuals with no history of cancer available from UKB, which were partitioned into three case-control analysis pillars in a ratio of approximately one TGCT case to ten unaffected UKB controls (ICR pillar: 986 TGCT cases/10 300 UKB controls; TCGA pillar: 150 TGCT cases/1500 UKB controls; and UKB pillar: 658 TGCT cases/6600 UKB controls; Supplementary Table 3) [13]. This study was conducted in accordance with the Code of Ethics of the World Medical Association (Declaration of Helsinki).

### 2.2 Alignment, variant calling, and quality control

**Fig. 1:**
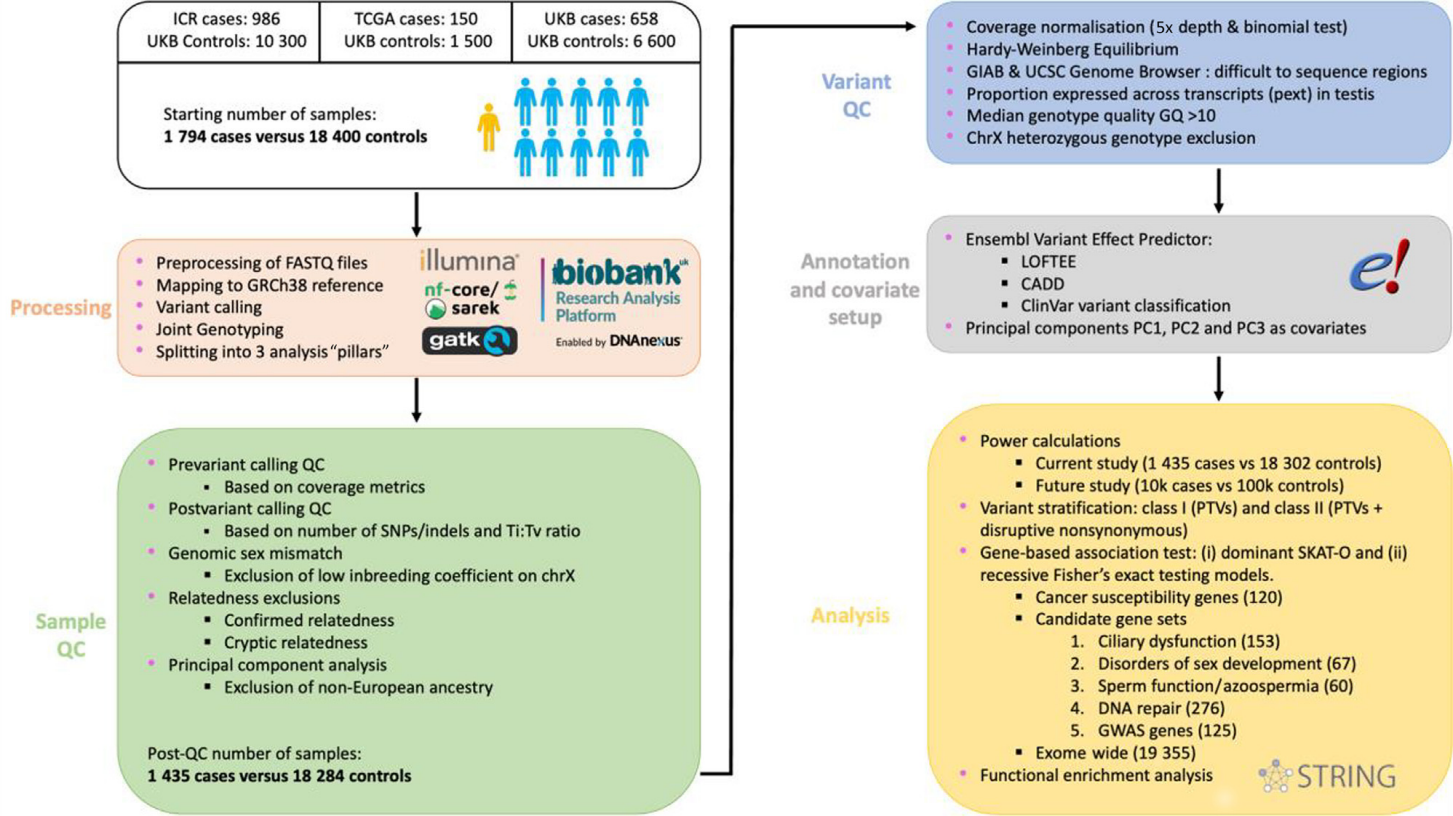
Workflow overview. A summary diagram displaying the process undertaken in the WES gene association analysis and highlighting the applied approach to ensure data accuracy and reliability. The workflow is divided into multiple stages, beginning with the processing of data, then proceeding with quality control on the sample and variant levels, followed by preparation of variant annotations and covariates (principal components PC1, PC2, and PC3), and culminating in the three rounds of gene-level association tests, functional enrichment, and power calculations. CADD = Combined Annotation Dependent Depletion; ChrX = X chromosome; GQ = genotype quality; GWAS = genome-wide association study; ICR = Institute of Cancer Research; LOFTEE = Loss-Of-Function Transcript Effect Estimator; PTV = protein-truncating variant; QC = quality control; SKAT-O = optimal sequence kernel association test; SNP = single nucleotide polymorphism; TCGA = The Cancer Genome Atlas; UKB = UK Biobank; WES = whole-exome sequencing.

The nf-core/sarek pipeline (v2.7) was used to map FASTQ files to GRCh38 for ICR and TCGA samples. Variants were called using DeepVariant (v1.4.0), generating per-sample VCF files. Individual case sample gVCF files were aggregated and jointly called with UKB controls using GLNexus (v1.0.4), and then divided into pillars as described. Per-pillar quality control (QC) steps were performed at the sample (filtering against genomic sex mismatch, kinship, and non-European ancestry) and variant (filtering low-confidence calls) levels (Fig. 1, Supplementary Tables 4–6, and Supplementary Fig. 1). A total of 1435 cases (667 ICR, 119 TCGA, and 649 UKB) and 18 284 UKB controls were retained after QC (Supplementary Table 4).

### 2.3 Gene set curation and analyses

Our TGCT susceptibility gene discovery analysis comprised three stages: Firstly, we evaluated the contribution of variants in 120 established cancer susceptibility genes (CSGs; Supplementary Table 7) [14], [15], 92 under a dominant model (including X- and Y-linked genes) and 44 under a recessive model of inheritance. Secondly, we evaluated variants in genes within five prespecified pathways with strong candidacy based on their role in TGCT tumorigenesis (Supplementary Table 8) [4], [7], [11], [16], [17]. Thirdly, we performed an exome-wide analysis considering 19 355 protein-coding genes.

### 2.4 Variant stratification

In the CSG analysis, we focused on variants classified as clinically pathogenic (high-confidence [HC] LoF variants, as predicted by Loss-Of-Function Transcript Effect Estimator [LOFTEE] and/or assigned as pathogenic/likely pathogenic on ClinVar [one or more star review status; Supplementary Table 9]). Subsequently, for candidate gene set and exome-wide analyses, variants were stratified into two tiered classes: (1) HC LoF and (2) HC LoF plus nonsynonymous (including base substitutions and inframe indels) with a Combined Annotation Dependent Depletion (CADD) v1.6 PHRED score of ≥20 (Supplementary Tables 10 and 11).

### 2.5 Statistical analyses

Gene-level association testing assuming a dominant model (one or more qualifying variants per gene) was conducted using SAIGE-GENE+ [18] and the optimal sequence kernel association test (SKAT-O; Supplementary Fig. 2) [19] with minor allele frequency (MAF) weighting (Supplementary Fig. 3). The recessive model involved retaining variants with an MAF of <1% and comparing the frequency of cases and controls (two or more qualifying variants per gene) using Fisher’s exact test. Resulting *p* values from each of the three analysis pillars were combined using Stouffer’s method [20], while applying weights based on case sample size and gene coverage (proportion of sites >5×; Supplementary Table 12). Multiple testing was sequentially corrected for each round of analysis using the Bonferroni method.

### 2.6 Functional enrichment analysis

A ranked functional enrichment analysis was performed on *p*-value results using STRING (version 12.0) [21]. An initial analysis included all three gene ontology (GO) groups (biological process, cellular component, and molecular function), before subsequent incorporation of InterPro, Pfam, Reactome, and STRING clusters [22], [23], [24].

### 2.7 Power analyses

We ran simulations for various combinations of combined MAF in controls (ranging from 0.0001 to 0.01) and gene-level effect size (odds ratios [ORs] of 2, 3, 4, 5, 8, and 10). Using the same sample size as the primary analysis, a similar SAIGE-GENE+ SKAT-O test was executed 10 000 times per frequency/OR combination. Statistical power was determined by assessing the proportion of simulated genes that crossed the exome-wide significance threshold (*p* = 6.60 × 10^–7^).

## 3 Results

### 3.1 Overview

Following QC exclusions, the final combined dataset comprised 1435 men with TGCTs and 18 284 cancer-free male controls (Table 1). The epidemiological profile of TGCT cases broadly reflected reported distributions regarding pathology, with some variation observed in age at diagnosis (interquartile range 30–45) [25]. Of the cases, 8.6% had a family history of TGCTs (compared with a reported population background rate of 1.4%) [26] reflecting proactive recruitment of familial cases in the ICR series.

**Table 1:** Clinical characteristics of cases following QC exclusions

	ICR	TCGA	UKB	Combined
Number of TGCT cases (%)	667 (46.48)	119 (8.29)	649 (45.23)	1435 (100)
Age				
Median	34	31	41	38
IQR	27–41	26–38	34–48	30–45
Tumour histology, *n* (%)				
Seminoma	253 (37.93)	52 (43.70)	440 (67.80)	745 (51.92)
Nonseminoma	148 (22.19)	31 (26.05)	171 (26.35)	350 (24.39)
Mixed	51 (7.65)	22 (18.49)	38 (5.86)	111 (7.74)
Unspecified	215 (32.23)	14 (11.76)	0 (0)	229 (15.96)
Family history of TGCT, *n* (%)				
Yes	123 (18.44)	0 (0)	0 (0)	123 (8.57)
None/not known	544 (81.56)	119 (100)	649 (100)	1312 (91.43)

ICR = Institute of Cancer Research; IQR = interquartile range; QC = quality control; TCGA = The Cancer Genome Atlas; UKB = UK Biobank.

### 3.2 Power analysis

Under a dominant model, we had good (>80%) power to identify associations exome-wide associations for genes with high-risk effects (OR >5), assuming that the collective frequency of contributing rare variants in controls (MAF_combined_) was >0.001 (0.1%; Supplementary Fig. 4A). For genes of more intermediate effect sizes (OR = 4), power was also >80%, provided that MAF_combined_ was >0.003 (0.3%). Power was, however, reduced for genes conferring more modest effect sizes: for effect size of OR = 3, power was <80% for any MAF_combined_ <0.006 (0.6%). For a gene of OR = 2, power was below 40% even for a gene of MAF_combined_ = 0.01 (1%). Power was, unsurprisingly, even lower under a recessive model (Supplementary Fig. 4B) with power to detect contributing rare variants in both alleles of >80% achieved only when OR = 10 and MAF_combined is_ ≥0.01 (1%). Considering power for a hypothetical future analysis comprising 10 000 TGCT cases and 100 000 controls (Supplementary Fig. 4C and 4D), the power under both models is enhanced greatly for the detection of hypothetical gene associations across most ORs and control allele frequencies. For the detection of exome-wide associations for genes of modest effect size (OR = 2), power is over 80% provided that MAF_combined_ is >0.006 (0.6%) under a dominant model. Power for moderate- to high-risk effects (OR >4) would be good (>80%) under a recessive model down to a MAF_combined_ of 0.008 (0.8%).

### 3.3 Pathogenic variants in CSGs

**Fig. 2:**
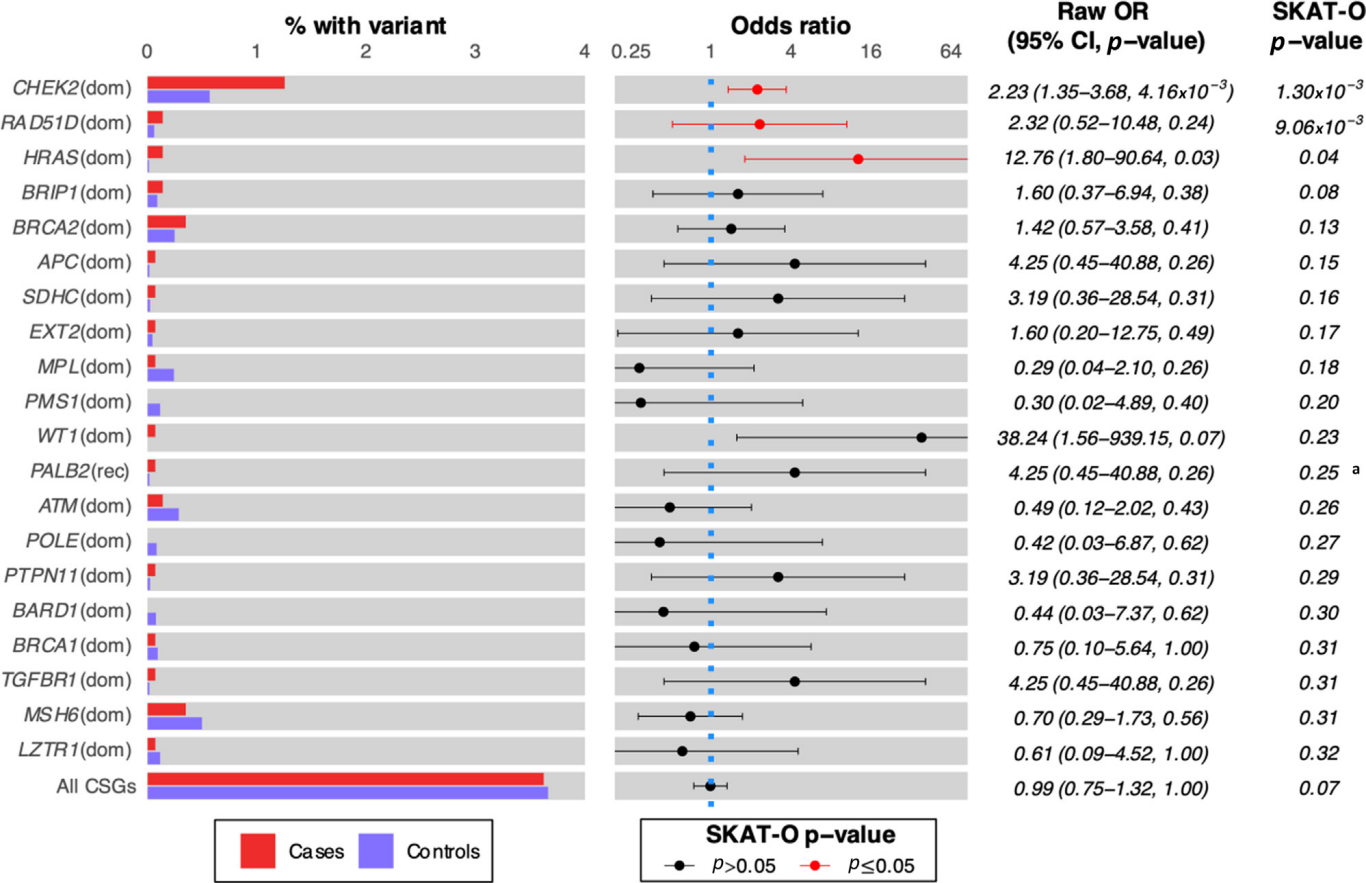
Frequency of CSG pathogenic variants in TGCT cases and controls. Frequency of carriers of high-confidence loss of function variants (HC LoF, as defined by LOFTEE) plus pathogenic/likely pathogenic variants (defined by one or more star review status ClinVar entries) in 120 established CSGs (70 dominantly acting, 28 recessive, 16 dominant + recessive, five X linked, and one Y linked) between cases and controls. An analysis was carried out under both a dominant (one or more qualifying variants per gene amongst 92 CSGs, including X- and Y-linked genes) and a recessive (two or more qualifying variants per gene amongst 44 CSGs) model. The relevant model from which the *p* value (as quantified by SKAT-O) and raw odds ratio (OR) are derived is listed in brackets after the gene name. ORs and corresponding *p* values (quantified by a Fisher’s exact test) were calculated from raw counts of individuals harbouring the requisite number of qualifying variants. The left panel shows the percentage of cases/controls harbouring qualifying variants. The right panel shows the corresponding OR and the 95% confidence interval (CI) on a base-4 log scale, with the 95% confidence limits truncated for some variants with wide intervals. A vertical blue dotted line is drawn at the baseline OR of 1. The genes are ordered by SKAT-O *p* values, and only the top 20 ranking genes have been shown. The Bonferroni-corrected *p*-value significance threshold is set at 3.68 × 10^–4^ for the curated gene sets (141 tests). The *p* value for all CSGs was calculated with a Cauchy combination of the *p* values from all gene-level SKAT-O *p* values under both dominant and recessive models. CI = confidence interval; CSG = cancer susceptibility gene; dom = dominant; LOFTEE = Loss-Of-Function Transcript Effect Estimator; OR = odds ratio; rec = recessive; SKAT-O = optimal sequence kernel association test; TGCT = testicular germ cell tumour. ^a^ Fisher’s exact *p* value is presented instead of SKAT-O for PALB2 only due to a recessive model of inheritance.

We first analysed 120 established CSGs (70 autosomal dominant, 28 autosomal recessive, 16 autosomal dominant + recessive, five X linked, and one Y linked) for an association with TGCT [15], [27], [28]. Examining for the presence of “clinically pathogenic” qualifying variants (see the Patients and methods section) in dominant and recessive genes (one and two variants, respectively), we did not identify any gene to be significantly associated with TGCTs after multiple testing corrections (significance threshold of *p* ≤ 3.68 × 10^–4^; Fig. 2 and Supplementary Table 13). The most significant association was for pathogenic variants in *CHEK2*: 18/1435 in cases (1.25%) and 104/18 284 (0.57%) in controls (OR = 2.23 [95% confidence interval {CI} 1.35–3.68, *p* = 1.30 × 10^–3^]); the c.1100delC founder variant was identified in 12/1435 cases and 78/18 284 controls (OR = 1.95 [95% CI 1.24–3.09]; Supplementary Table 14) [10]. While clonal haematopoiesis for *CHEK2* variants has previously been reported in several cancer types, we detected no systematic differences in variant allele frequency (VAF) in our data between cases and controls (*p* = 0.803) [29]. There was likewise no evidence of systematic differences in VAF for *HRAS* (*p* = 0.788), another gene nominally associated, in which clonal haematopoiesis has been reported [30]. Across all 120 CSGs, the proportion of men carrying a qualifying clinically pathogenic variant in a dominant CSG (or two in a recessive CSG) was not significantly elevated in cases (52/1435 or 3.62%) compared with controls (670/18 284 or 3.66%; OR = 0.99 [95% CI 0.75–1.32]), suggesting that minimal unexplained residual pleiotropy for TGCTs is accounted for by these genes.

### 3.4 Gene-level association of candidate gene sets

We next prioritised genes plausibly associated with TGCTs based on biological candidacy, examining four functional groupings—ciliary dysfunction (153 genes), disorders of sex development (67 genes), sperm function/azoospermia (60 genes), and DNA repair (276 genes), and a fifth group comprising genes in linkage disequilibrium with GWAS-identified loci (r^2^ > 0.4; 125 genes), totalling 655 unique genes [4], [11]. Considering a dominant model for both autosomal and X chromosome genes, collapsing at gene level, we compared the frequency in cases and controls of class I (HC LoF variants) and class II (HC LoF variants plus nonsynonymous with CADD ≥20) variants in each of these 655 genes using SKAT-O testing to weight for variant frequency. After multiple testing corrections (Bonferroni-corrected *p* < 1.78 × 10^–5^), no significant association was observed for either class of qualifying variants (Table 2 and Supplementary Table 15). We next evaluated these 655 genes under a recessive model for classes I and II, excluding X-linked genes, comparing the frequency of cases and controls in which two qualifying variants were present. Again, after cumulative multiple testing corrections (Bonferroni-corrected *p* = 1.78 × 10^–5^), no significant associations were identified (Supplementary Table 16).

**Table 2:** Association analysis of TGCTs with candidate gene sets under dominant and recessive models

Candidate gene set	Dominant model				Recessive model			
	Class I: HC LoF		Class II (HC LoF plus nonsynonymous CADD ≥20)		Class I: HC LoF		Class II (HC LoF plus nonsynonymous CADD ≥20)	
	Gene	*p* value	Gene	*p* value	Gene	*p* value	Gene	*p* value
Ciliary dysfunction	*DCDC2* *ANKS6* *IFT140* *SPATA7* *NEK1*	5.10 × 10^–2^ 5.45 × 10^–2^ 6.41 × 10^–2^ 0.10 0.11	*CEP290* *NPHP1* *IQCE* *CEP83* *VPS13B*	6.82 × 10^–4^ 1.59 × 10^–3^ 2.88 × 10^–3^ 5.42 × 10^–3^ 1.26 × 10^–2^	– – – – –	– – – – –	*VPS13B* *IMPDH1* *PIBF1* *DDX59* *TOGARAM1*	6.07 × 10^–2^ 0.15 0.30 0.33 0.44
Sex development	*CYP11B1* *MAP3K1* *CYP11A1* *HSD17B3* *ESR2*	5.18 × 10^–2^ 6.91 × 10^–2^ 0.14 0.15 0.16	*NR3C1* *TSPYL1* *MCM5* *CYP11B1* *ATP6V0A4*	3.19 × 10^–3^ 1.12 × 10^–2^ 1.72 × 10^–2^ 2.38 × 10^–2^ 3.06 × 10^–2^	– – – – –	– – – – –	*TOE1* *PPP1R12A* *–* *–* *–*	0.25 0.47 – – –
Sperm function/azoospermia	*M1AP* *SHOC1* *TERB2* *CFTR* *MAJIN*	8.30 × 10^–3^ 4.87 × 10^–2^ 5.39 × 10^–2^ 7.15 × 10^–2^ 0.11	*ATM* *MSH4* *M1AP* *CFTR* *SLC22A5*	5.34 × 10^–3^ 1.10 × 10^–2^ 1.27 × 10^–2^ 1.47 × 10^–2^ 2.21 × 10^–2^	– – – – –	– – – – –	*DNAH1* *CFTR* *TEX14* *ATM* *–*	8.09 × 10^–2^ 0.20 0.56 0.66 –
DNA repair	*CHEK2* *RAD51D* *CHEK1* *RAD54L* *SMUG1*	3.35 × 10^–4^ 9.06 × 10^–3^ 2.64 × 10^–2^ 2.91 × 10^–2^ 3.02 × 10^–2^	*ERCC6* *CHEK2* *INO80* *BLM* *XRCC1*	9.10 × 10^–4^ 1.47 × 10^–3^ 2.66 × 10^–3^ 2.87 × 10^–3^ 3.03 × 10^–3^	*APLF* *PALB2* *RAD52* *NUDT15* *–*	0.20 0.25 0.32 0.60 –	*RECQL* *ATR* *PALB2* *APLF* *EXO1*	0.16 0.18 0.18 0.20 0.23
GWAS-related genes	*PCNT* *SEPTIN4* *MAD1L1* *CDKL2* *HEATR3*	2.94 × 10^–2^ 3.65 × 10^–2^ 4.10 × 10^–2^ 6.99 × 10^–2^ 8.34 × 10^–2^	*SMARCAD1* *MTMR4* *CLPTM1L* *LCTL* *HEATR3*	1.19 × 10^–2^ 1.29 × 10^–2^ 1.35 × 10^–2^ 1.36 × 10^–2^ 1.56 × 10^–2^	*PCNT* *RAD52* *–* – –	0.24 0.32 – – –	*WNK1* *PLBD1* *HEATR3* *PRTG* *NTAN1*	0.19 0.21 0.21 0.26 0.29

CADD = Combined Annotation Dependent Depletion; GWAS = genome-wide association study; HC = high confidence; LoF = loss of function; SKAT-O = optimal sequence kernel association test; SNP = single-nucleotide polymorphism; TGCT = testicular germ cell tumour.

Shown are the five highest-ranking genes by *p* values from an association analysis of precurated candidate gene sets relating to ciliary functions, disorders of sex development, sperm function/azoospermia, and DNA repair, as well as genes of correlation r^2^ >0.4 with the most significant candidate SNPs from GWAS studies. A SKAT-O analysis in the dominant model and Fisher’s exact test in the recessive model were performed for two variant classes: (1) class I: HC LoF (determined by LOFTEE annotation) and (2) class II: HC LoF plus nonsynonymous with CADD PHRED score of ≥20. Results are shown for the meta-analysis using the Stouffer's method of *p*-value combination. The Bonferroni-corrected significance threshold is set at 1.78 × 10^–5^ for the curated gene sets (2810 cumulative tests).

### 3.5 Exome-wide analysis

Following candidate gene analyses, we conducted an exome-wide gene association analysis (19 355 genes). Correcting cumulatively for multiple testing, no significant association was observed for either class I or class II variants in any gene under either dominant or recessive models of inheritance (Bonferroni-corrected *p*-value threshold = 6.60 × 10^–7^; Table 3, Supplementary Fig. 5, and Supplementary Table 17). Furthermore, conducting association subanalyses restricted to (1) 745 seminoma samples and (2) 461 nonseminoma/mixed samples yielded no significant histology-specific gene associations under a dominant model (Supplementary Table 18).

**Table 3:** Exome-wide TGCT association analysis assuming each of a dominant and recessive model

Dominant model				Recessive model			
Class I: HC LoF		Class II (HC LoF plus nonsynonymous CADD ≥20)		Class I: HC LoF		Class II (HC LoF plus nonsynonymous CADD ≥20)	
Gene	*p* value	Gene	*p* value	Gene	*p* value	Gene	*p* value
*PLA2G3*	6.07 × 10^–5^	*RNPC3*	1.61 × 10^–5^	*AVIL*	1.22 × 10^–2^	*RSPH10B2*	9.19 × 10^–3^
*INCA1*	1.74 × 10^–4^	*FAT4*	2.98 × 10^–5^	*SERPINA3*	3.38 × 10^–2^	*ADGRV1*	9.39 × 10^–3^
*COL27A1*	1.95 × 10^–4^	*DGLUCY*	5.82 × 10^–5^	*NME9*	4.00 × 10^–2^	*RANBP2*	1.10 × 10^–2^
*C4orf54*	3.16 × 10^–4^	*DHRS7C*	9.24 × 10^–5^	*ART5*	5.29 × 10^–2^	*LRIG2*	1.24 × 10^–2^
*CHEK2*	3.35 × 10^–4^	*MYO1C*	9.73 × 10^–5^	*ATIC*	0.11	*IFNE*	1.39 × 10^–2^
*GSTM4*	4.88 × 10^–4^	*CARD10*	1.13 × 10^–4^	*PLA2G3*	0.14	*BTN2A2*	1.81 × 10^–2^
*SLCO1B3*	6.63 × 10^–4^	*EEF1AKMT1*	1.23 × 10^–4^	*DBF4B*	0.16	*HABP2*	2.09 × 10^–2^
*ACP3*	8.08 × 10^–4^	*ETFBKMT*	1.34 × 10^–4^	*APLF*	0.20	*AMPD3*	2.26 × 10^–2^
*GUCY1A1*	1.23 × 10^–3^	*ATP11A*	1.60 × 10^–4^	*ADAMTS7*	0.20	*PLCG2*	2.30 × 10^–2^
*FGF11*	1.68 × 10^–3^	*PLA2G3*	1.71 × 10^–4^	*KIAA1328*	0.22	*MAGI2*	2.44 × 10^–2^

CADD = Combined Annotation Dependent Depletion; HC = high confidence; LoF = loss of function; SKAT-O = optimal sequence kernel association test; TGCT = testicular germ cell tumour.

The table presents the ten highest-ranking genes by *p* value from an association analysis of 19 355 genes across the whole exome. A SKAT-O analysis in the dominant model and Fisher’s exact test in the recessive model were performed for two variant classes: (1) class I: HC LoF and (2) class II: HC LoF plus nonsynonymous with CADD ≥20. Results are shown for the meta-analysis using the Stouffer's method of *p*-value combination. The Bonferroni-corrected *p*-value threshold of significance is set at 6.60 × 10^–7^ (75 760 cumulative tests).

### 3.6 Functional enrichment analysis

We next moved from per-gene to per-gene-set analyses based on the sets of genes grouped by commonality of function. We examined 22 668 predefined functional groupings of genes defined within STRING databases from GO terms and protein domains. These functional enrichment analyses were performed using the ordered *p* values derived under each of the dominant and recessive models of the exome-wide analysis and for both class I/II variants, thus comprising four independent analyses.

Four from the 22 668 gene groups were statistically significant when applying standard thresholds of a false discovery rate (FDR) of <0.01 and an enrichment score of >2 (Supplementary Fig. 6 and Supplementary material), all of which are related to microtubular and ciliary function (4/260 microtubular/ciliary pathways vs 0/22 408 other pathways). Bringing all genes in the 260 microtubular/ciliary pathways into one umbrella functional grouping, we also observe evidence of enrichment relative to all other gene sets (*p* = 1.69 × 10^–8^). The association was replicated using 48 593 gene groupings assembled via four different gene-set databases, wherein 27/38 gene groupings exhibiting significant enrichment (FDR <0.01 and enrichment score >2) were amongst the 476 related to microtubular/ciliary function (27/476 vs 11/48 593, *p* = 2.28 × 10^–46^; Supplementary Table 19).

## 4 Discussion

We report a meta-analysis of three datasets constituting 19 719 samples (1435 TGCT cases and 18 284 male controls), which, to our knowledge, represents the largest TGCT exome discovery analysis reported to date. Rather than the standard approach of a fixed frequency cut-off, we applied a SKAT-O approach for collapsing qualifying disruptive variants by gene, incorporating a weighting based on variant frequency that better captures gene associations spanning the continuum of variant frequencies [18].

Given that TGCTs have been highlighted for the strength and biological candidacy of their common variant associations, this strategy was intended to provide better facility to identify genes for which an association might include more common, as well as rarer, variants [3], [31], [32]. We optimised our power for discovery by focusing first on the sets of genes potentially functionally implicated in TGCTs, before proceeding to an exome-wide analysis. Correcting sequentially for multiple testing, we found no significant association for any individual genes in analyses of disruptive variants at the gene level based on candidacy related to (1) established cancer susceptibility, (2) ciliary dysfunction, (3) disorders of sex development, (4) spermatogenesis/azoospermia, (5) DNA repair, (6) GWAS candidate genes, and finally (7) exome wide. While the previously observed higher relative risk between brothers compared with that in a father-son duo supports the hypothesis of an X-linked or recessive inheritance in TGCTs, our results did not identify any genes associated with TGCTs under either of these inheritance models.

Although we found no statistically significant associations with TGCTs amongst 120 established CSGs to suggest unexplained pleiotropy for TGCTs, the strongest association was for *CHEK2.* This replicated an association reported previously in a candidate gene study, which is highly plausible given the growing evidence for pleiomorphic moderate-risk association of *CHEK2* across multiple cancer types [9], [10]. However, there was no replication of candidate TGCT-associated genes identified in earlier exome-wide discovery analyses; for the ten genes (LoF variants) reported recently as significantly associated by Pyle et al [11] in their analysis of 228 TGCT familial probands, *p* values were >0.01 in each of the four analyses (class I/class II, dominant/recessive). Functional gene-set enrichment analysis implicated gene pathways related to microtubule/ciliary function as associated with TGCTs; genes in these pathways have also been identified at GWAS-identified TGCT loci [1], [4], [33]. However, individual gene-level associations were not significant for any gene, including *DNAAF1,* the microtubule/ciliary gene identified in our earlier familial whole-exome sequencing TGCT segregation-based analysis [7].

This analysis focused only on small variants: structural variants, such as deletions and duplications, were not investigated due to inherent challenges in their identification using short-read NGS data. Few studies directed at identifying susceptibility driven by large structural events exome wide have been carried out on TGCT cohorts [34]; however, germline structural variations in other cancers have been reported previously, most notably in CSGs [35]. A lack of paired controls for the ICR and TCGA studies necessitated the use of UKB as a universal source of controls across case series; recruitment to UKB has been argued to bias towards healthier individuals with potential contribution to this phenomenon via our use of cancer-free controls [36]. As is typical for meta-analysis of disparate datasets, there was heterogeneity in technical aspects of sequencing, including capture methods and sequencing quality. This heterogeneity was mitigated with a rigorous sample-level QC, resulting in exclusion of case samples of lower sequencing quality, as well as a gene coverage weighting for the combination of results. Another potential limitation is the absence of diagnostic evaluation for the control group, which could possibly include individuals with indolent/undiagnosed TGCTs. However, given that the majority of UKB controls were recruited at relatively older age and TGCT typically presents in younger individuals, the likelihood of an undiagnosed TGCT is minimal.

## 5 Conclusions

Exposition of the approximate half of the sizeable heritability of testicular cancer ascibable to rare variants remains frustratingly elusive [37]. Our study was much better powered (approximately eight-fold of the total sample number and ∼1.5-fold of the number of cases) than the previous largest TGCT exome discovery analysis that we reported in 2018, comprising 972 cases and 1644 controls [8]. Lack of replication in this exome-wide analysis of previously reported association signals highlights the widely recognised challenge of this field, namely, the potential for first reporting of inflated or erroneous associations resulting in type 1 errors (Winner’s curse), followed by the punitive impact of correction for multiple testing in replication experiments, potentially resulting in type 2 errors especially where variant effect sizes/frequencies are modest. Indeed, power calculations indicate that TGCT-associated coding variants are likely to be either extremely infrequent and/or of modest effect size (OR = 4), consistent with a highly polygenic architecture underpinning the sizeable heritable component of TGCT [38], necessitating substantially larger studies to better reveal these gene associations.

  **Author contributions:** Clare Turnbull had full access to all the data in the study and takes responsibility for the integrity of the data and the accuracy of the data analysis.

  Study concept and design: Kuzbari, Loveday, Garrett, Turnbull.

Acquisition of data: Kuzbari, Wade, Broderick, Litchfield.

Analysis and interpretation of data: Kuzbari, Wade, Rowlands, Turnbull, Loveday, Garrett, Choi.

Drafting of the manuscript: Kuzbari, Rowlands, Garrett, Turnbull, Loveday.

*Critical revision of the manuscript for important intellectual content*: Turnbull, Rowland.

Statistical analysis: Kuzbari, Turnbull.

Obtaining funding: Turnbull, Houlston, Huddart.

Administrative, technical, or material support: Torr.

Supervision: Turnbull, Rowlands, Loveday.

Other: Patient recruitment: Turnbull, Huddart, Reid.

  **Financial disclosures:** Clare Turnbull certifies that all conflicts of interest, including specific financial interests and relationships and affiliations relevant to the subject matter or materials discussed in the manuscript (eg, employment/affiliation, grants or funding, consultancies, honoraria, stock ownership or options, expert testimony, royalties, or patents filed, received, or pending), are the following: None.

  **Funding/Support and role of the sponsor:** This work was supported by the Movember Foundation and the ICR. The ICR played a role in the collection of data and the funding of Zeid Kuzbari’s PhD studentship. Charlie F. Rowlands, Isaac Wade, Alice Garrett, Chey Loveday, Subin Choi, and Beth Torr are supported by Cancer Research UK (CRUK) Catalyst Award CanGene-CanVar (C61296/A27223).

  **Acknowledgements:** We thank the patients and their clinicians for participation in this study. We acknowledge the International Testicular Cancer Linkage Consortium (ITCLC) and the UK Testicular Cancer Collaboration. We acknowledge the National Health Service funding to the National Institute for Health Research Biomedical Research Centre. We acknowledge D. Timothy Bishop, Michael Stratton, Gillian Crockford, Rachel Linger, Darshna Dudakia, and Elizabeth Rapley for coordination of early sample recruitment, and all members of the ITCLC. We acknowledge the facilities and expertise of the Cancer Genetics Core Laboratory Facility and Cancer Genetics Sequencing Facility at the ICR directed by Nazneen Rahman, supported by Sheila Seal, Anthony Renwick, Emma Ramsey, and Elise Ruark.
