# Supplementary material for: Meta-analysis of Germline Whole-exome Sequencing in 1435 Cases of Testicular Germ Cell Tumour to Evaluate Disruptive Mutations Under Dominant, Recessive, and X-linked Inheritance Models
Source: Eur Urol Open Sci. 2025 Feb 13;73:51–9. doi: 10.1016/j.euros.2025.01.015 (PMC11875187; doi:10.1016/j.euros.2025.01.015)
Supplement: Supplementary Data 1 [file mmc1.docx]

# **Supplementary material**

# Supplementary Notes

**Supplementary Note 1.** The UK Testicular Cancer Collaboration (UKTCC)

| **Principal investigator** | **Study centre** | **Study centre address** |
| --- | --- | --- |
| Prof. Gordon Rustin | Mount Vernon Hospital | Mount Vernon Cancer Centre, Rickmansworth Road, Northwood, Middlesex, HA6 2RN |
| Dr. Srihari | Royal Shrewsbury Hospital | Trials Unit, Oncology Department, Mytton Oak Road, Shrewsbury, SY3 8XB |
| Dr. David Cole | Great Western Hospital | 3rd Floor, Osprey Unit, Swindon, Wilts, SN3 6BB |
| Dr. Colin Askill & Dr. Gianfilippo Bertelli | Singleton Hospital and Morriston Hospital | SWW Cancer Institute, Sketty, Swansea, SA2 8QA |
| Dr. James Barber | Velindre Hospital | Clinical Trials Unit, Velindre Cancer Centre, Velindre Road, Whitchurch, Cardiff CF14 2TL |
| Dr. Ed Gilby | Royal United Hospital | Dept of Oncology and Haematology, Combe Park, Bath, BA1 3NG |
| Dr. Robert Huddart | Royal Marsden Hospital Sutton | Downs Rd, Sutton, SM2 5PT |
| Dr. Jeff White | Beatson Oncology Centre | Beatson West of Scotland Cancer Centre, 1053 Great Western Road, Glasgow, G11 0YN |
| Dr. Jeremy Braybrooke | Bristol Haematology & Oncology Centre | United Bristol Healthcare NHS trust, Horfield Rd, Bristol, BS2 8ED |
| Dr. M Leahy & Dr. R. Welch | Christie Hospital | Wilmslow Road, Withington, Manchester, M20 4BX |
| Dr. P. Chakraborti | Derbyshire Royal Infirmary | Derby Hospitals NHS Trust, London Road, Derby, DE1 2QY |
| Dr. J. Joffe | St James Hospital Leeds | Dept of Medical Oncology, Leeds, LS9 7TF |
| Dr. Richard Brown | Wexham Park Hospital | Cancer Clinical Trials, John Ulster Post Grad Centre, Slough, Berks, SL2 4HL |
| Dr. Guy Faust | Leicester Royal Infirmary | LNR Cancer Research Network, Knighton St, Leicester LE1 5WW |
| Dr. Peter Simmonds | Southampton General Hospital | Cancer Care Directorate, Medical Oncology, Mailpoint 306, Southampton General Hospital, Tremona Rd, SO16 6YD |
| Dr. Danish Mazhar | Addenbrookes Hospital | Addenbrookes Hospital, Cambridge Clinical Trials Centre, Oncology Clinical Trials, (S4) Box 279, Hills Rd, CB2 0QQ |
| Dr. A. Stockdale, Dr. D. Hrouda, & Dr. C. Humber | University Hospital Walsgrave | Arden Cancer Centre, West Wing, UHCW NHS trust, Clifford Bridge Rd, Coventry, CV2 2DX |
| Dr. Wiebke Appel | Royal Preston Hospital | Dept of Oncology, Royal Preston Hospital, Sharoe Green Lane North, Fulwood Preston, PR2 9HT |
| Dr. Anne Hong | Royal Devon & Exeter | Exeter Oncology Centre, Royal Devon and Exeter Hospital, Barrack Rd, Exeter EX2 5DW |
| Dr. Howard | Western General Hospital | Scottish Cancer Research Network, Oncology Admin Corridor, Edinburgh Cancer Centre, Western General Hospital, Crewe Rd South, Edinburgh, EH4 2XU |
| Dr Fiona Douglas | Freeman Hospital | Clinical Trials Unit, Newcastle General Hospital, Westgate Rd, Newcastle-upon Tyne, NE4 6BE |
| Dr. David Bllomfield | Royal Sussex County Hospital | Brighton and Sussex University Hospitals, The Sussex Cancer Centre, The Royal Sussex County Hospital, Eastern Road, Brighton, BN2 5BE |
| Dr. Mohammad Butt | Castle Hill Hospital | Castle Hill Hospital, Castle Road, Cottingham HU16 5JQ |
| Dr Kay Kelly | Raigmore Hospital | Raigmore Hospital, Old Perth Road, Inverness, IV2 3UJ |
| Dr. R. Mehra | New Cross Hospital | Greater Midlands Cancer Research Network, The Chestnuts, The Royal Wolverhampton Hospitals, New Cross Hospital NHS Trust, Wednesfield Road, Wolverhampton, WV10 0QP |
| Dr. Richard Brown & Dr. Paul Rogers | Royal Berkshire Hospital | Royal Berkshire Hospital, Berkshire Cancer Centre, London Road, Reading, Berkshire, RG1 5AN |
| Dr. P. Chakraborti | Queen’s Hospital Burton | Queen’s Hospital, Burton upon Trent, Belvedere Road, Burton, DE13 0RB |
| Dr. Matthew Hatton | Weston Park Hospital | Consultant Clinical Radiologist. Sheffield Teaching Hospitals NHS Foundation Trust, 8 Beech Hill Road, Sheffield S10 2SB |
| Dr. Ivo Hennig | Nottingham City Hospital | Nottingham University Hospitals NHS Trust, City Hospital campus, Hucknall Road, Nottingham, NG5 1PB |
| Dr. J. McAteer | Belfast City Hospital | Northern Ireland Cancer Centre, Belfast City Hospital, Lisburn Rd, Belfast, BT9 7AB |
| Dr. Savage & Dr. Seckl | Charing Cross Hospital | Dept of Medical Oncology, Charing Cross, Fulham, Palace Rd, London W6 8RF |
| Dr. Joanna Gale | Portsmouth Haematology & Oncology Centre | Level B Queen Alexandra Hospital, Cosham, Portsmouth, PO6 3LY |
| Prof. Gordon Rustin | Hillingdon Hospital | R&D Office - Education Centre, Hillingdon Hospital, Pield Heath Road, Hillingdon, UB8 3NN |
| Prof. Peter Clark | Royal Liverpool & Broadgreen Hospitals | Prescot Street Liverpool, L78XP |
| Dr. Steve Woby | Royal Oldham Hospital/Pennine Acute Hospital | Roachdale Road Oldham OL1 2JH |
| Dr. Adrian Rathmell | James Cook Hospital | Middlesbrough TS4 3BW |
| Dr. Alan Lamont | Colchester/Essex County Hospital | Essex County |
| Dr. Guy Faust | Northampton General | Cliftonville, Northampton NN1 5BD |
| Dr. Naveed Sarwar | Basildon Hospital | Nethermayne Basildon Essex SS16 5NL |
| Prof. Nick Stuart | Glan Clwyd Hospital and Ysbyty Gwynedd | NW Cancer Treatment Centre, Glan Clwyd Hospital, LL18 5UJ |
| Dr. Simon Chowdhury | Guys & St Thomas’s | St. Thomas Street, London SE1 9RT |
| Dr. Sharon Beesley | Maidstone and Tunbridge NHS Trust | Maidstone Hospital, Hemitage Lane, Barming, Maidstone, Kent ME16 9QQ |
| Dr. Winkler | West Middlesex University Hospital | West Middlesex University Hospital NHS Trust, R&D Department, 4th Floor, East Wing, Twickenham Road, Isleworth, Middlesex TW7 6AF |
| Dr. Abdel Hamid | Broomfield Hospital | Broomfield Hospital, West Wing 2, Court Road, Broomfield, Chelmsford, Essex CM1 7ET |
| Dr. Sanjeev Pathak | Doncaster Royal Infirmary | Joint Research Office of Doncaster and Bassetlaw Hospitals NHS Foundation Trust, First Floor C Block, Doncaster Royal Infirmary, Armthorpe Road, Doncaster DN2 5LT |
| Dr. Krishnaswamy Madhavan | Southend University Hospital NHS Foundation Trust | Pittlewell Chase, Westcliff-On-Sea, Essex SSO 0RH |
| Dr. Martin Highley | Derriford Hospital (Plymouth) | Plymouth Hospitals NHS Trust, Derriford Hospital, Plymouth, PL6 8DH |
| Dr. Julian Money-Kyrle | Royal Surrey County Hospital | Royal Surrey County Hospital, St Lukes Cancer Centre, Egerton Road, Guildford, Surrey GU2 7XX |
| Dr. Cathryn Brock | Chelsea & Westminster Hospital NHS Foundation Trust | Chelsea & Westminster Hospital, Unit 101, 1st Floor, Harbour Yard, Chelsea Harbour, London SW10 0XD |
| Dr. Thiagarajan Sreenivasan | United Linconshire Hospitals NHS Trust | Lincoln County Hospital, Greetwell Road, Lincoln, LN2 5QY |
| Dr. Thiagarajan Sreenivasan | United Linconshire Hospitals NHS Trust | Pilgrim Hospital, Boston, Lincolnshire PE21 9QS |

**Supplementary Note 2**. International Testicular Cancer Linkage Consortium (ITCLC) centres from which samples were used in this study

| **Investigators** | **Study Centre** | **Study centre address** |
| --- | --- | --- |
| D. Timothy Bishop, Gillian P. Crockford | St James's University Hospital, Leeds | Cancer Research UK Clinical Centre, St James's University Hospital, Leeds, UK |
| Michael R. Stratton, Elizabeth A. Rapley, Rachel Linger, Sarah Hockley, Darshna Dudakia, Lola Johnson | The Institute Of Cancer Research | Section of Cancer Genetics, Institute Of Cancer Research, 15 Cotswold Road, Sutton, Surrey SM2 5NG, UK |
| Robert Huddart | The Institute Of Cancer Research/The Royal Marsden Hospital | Academic Radiotherapy Unit, Institute of Cancer Research, Sutton, Surrey, UK |
| Kathy Tucker, Michael Friedlander | New South Wales and Prince of Wales Hospital | Department of Medical Oncology, Division of Medicine, University of New South Wales and Prince of Wales Hospital Randwick, Sydney, Australia |
| Kelly-Anne Phillips | Peter MacCallum Cancer Centre | Department of Haematology and Medical Oncology, Peter MacCallum Cancer Centre, East Melbourne, Victoria, Australia |
| David Hogg, Michael A.S. Jewett | Princess Margaret Hospital | Princess Margaret Hospital and University of Toronto, Toronto, ON, Canada |
| Radka Lohynska | University Hospital | Department of Radiotherapy and Oncology, University Hospital, Prague, Czech Republic |
| Gedske Daugaard | Rigshospitalet | Department of Oncology, Rigshospitalet, Copenhagen, Denmark |
| Stéphane Richard | Centre Expert National Cancers Rares PREDIR | Génétique Oncologique EPHE-UMR 8125 Faculté de Médecine Paris-Sud and Service d’Urologie, CHU, Le Kremlin-Bicêtre, France |
| Agnes Chompret | Institut Gustave Roussy | Génétique Oncologique, Institut Gustave Roussy, Villejuif, France |
| Catherine Bonaïti-Pellié | Hôpital Paul Brousse | INSERM U535, Hôpital Paul Brousse, Villejuif, France |
| Axel Heidenreich | Phillips University | Department of Urological Oncology, Phillips University, Marburg, Germany |
| Peter Albers | Klinikum Kassel GmbH | Department of Urology, Klinikum Kassel GmbH, Moenchebergstr. 41-43, D-34125 Kassel, Germany |
| Edith Olah, Lajos Geczi, Istvan Bodrogi | National Institute of Oncology | Department of Molecular Genetics and Department of Chemotherapy, National Institute of Oncology, Budapest, Hungary |
| Wilma J. Ormiston, Peter A. Daly | St James's Hospital | Department of Medical Oncology, St James's Hospital, Dublin, Ireland |
| Parry Guilford | University of Otago | Cancer Genetics Laboratory, University of Otago, Dunedin, New Zealand |
| Sergei A. Tjulandin, Ludmila Liubchenko | N.N. Blokhin Russian Cancer Research Centre | Laboratory of Clinical Genetics, Institute of Clinical Oncology, N.N. Blokhin Russian Cancer Research Centre, Moscow, Russian Federation |
| Hans Stoll, Walter Weber | University Hospital | Medical Oncology, University Hospital, Basel, Switzerland |
| David Forman | Cookridge Hospital | Cancer Epidemiology, University of Leeds, Cookridge Hospital, Leeds LS16 6QB, UK |
| Timothy Oliver | Barts and The London Queen Mary’s School of Medicine | Department of Medical Oncology, Barts and The London Queen Mary’s School of Medicine, London, UK |
| Douglas F. Easton | Cancer Research UK | Cancer Research UK Genetic Epidemiology Unit, Strangeways Research Laboratory, Cambridge, UK |

# Supplementary Figures


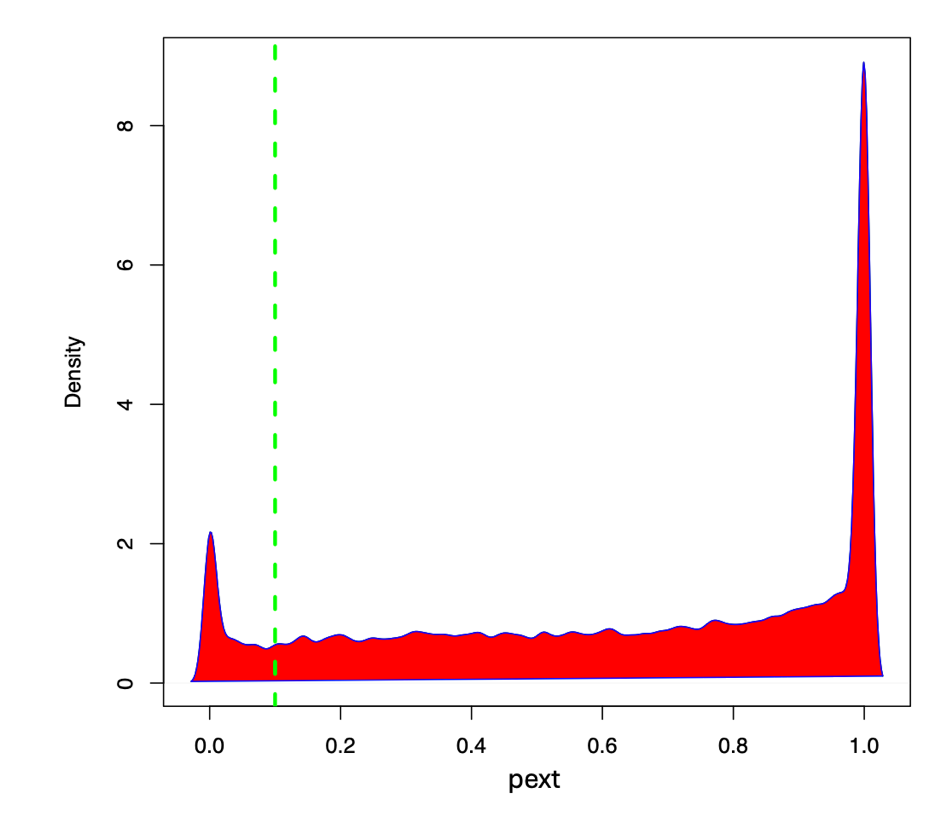


**Supplementary Figure 1. Proportion expressed across transcripts (pext) across all exonic sites in testis.** Density plot illustrating the distribution of pext values along the x-axis and their corresponding density on the y-axis within the context of testicular tissue. Pext values from GTEx represent the proportion of transcripts for a given gene for which a specific genomic site will be present in the final expressed transcript. The vertical green dotted line represents the critical cut-off point at 10%. Genomic sites with pext values below 10% in the testis have been excluded from the analysis as these are unlikely to have substantial biological impact in the testis.


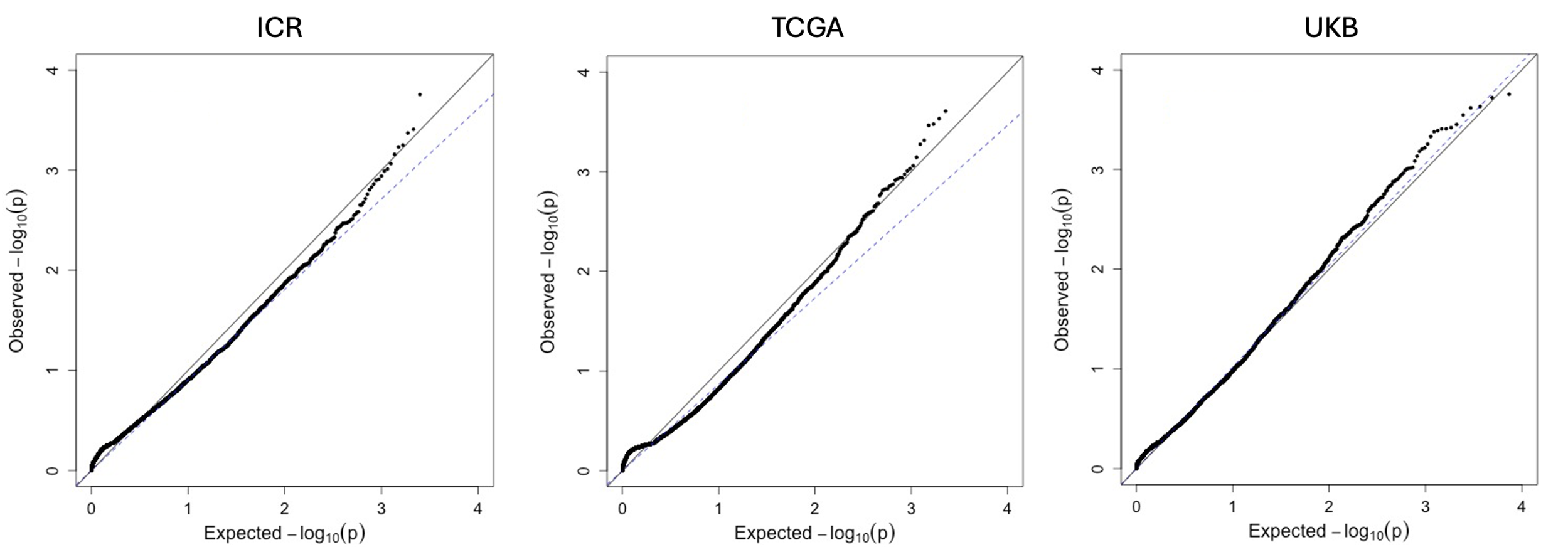


**Supplementary Figure 2. Quantile-quantile plot of *p*-values from SAIGE-GENE+ gene-level SKAT-O test of synonymous variants for each pillar of the meta-analysis.** The slope (λ_Δ95_) is used to assess the level of genomic inflation/deflation. When λ_Δ95_ <1, it indicates deflation, signifying that the observed data has a lower variance than expected under the null hypothesis. Conversely, when λ_Δ95_ >1, it suggests inflation, implying that the observed data exhibits higher variance than expected. The observed slopes for the ICR, TCGA, and UKB datasets were λ_Δ95_ = 0.90, 0.87, and 1.02, respectively. Spurious outlier genes with an observed -log10(p) >4 were excluded from the plots for scale.


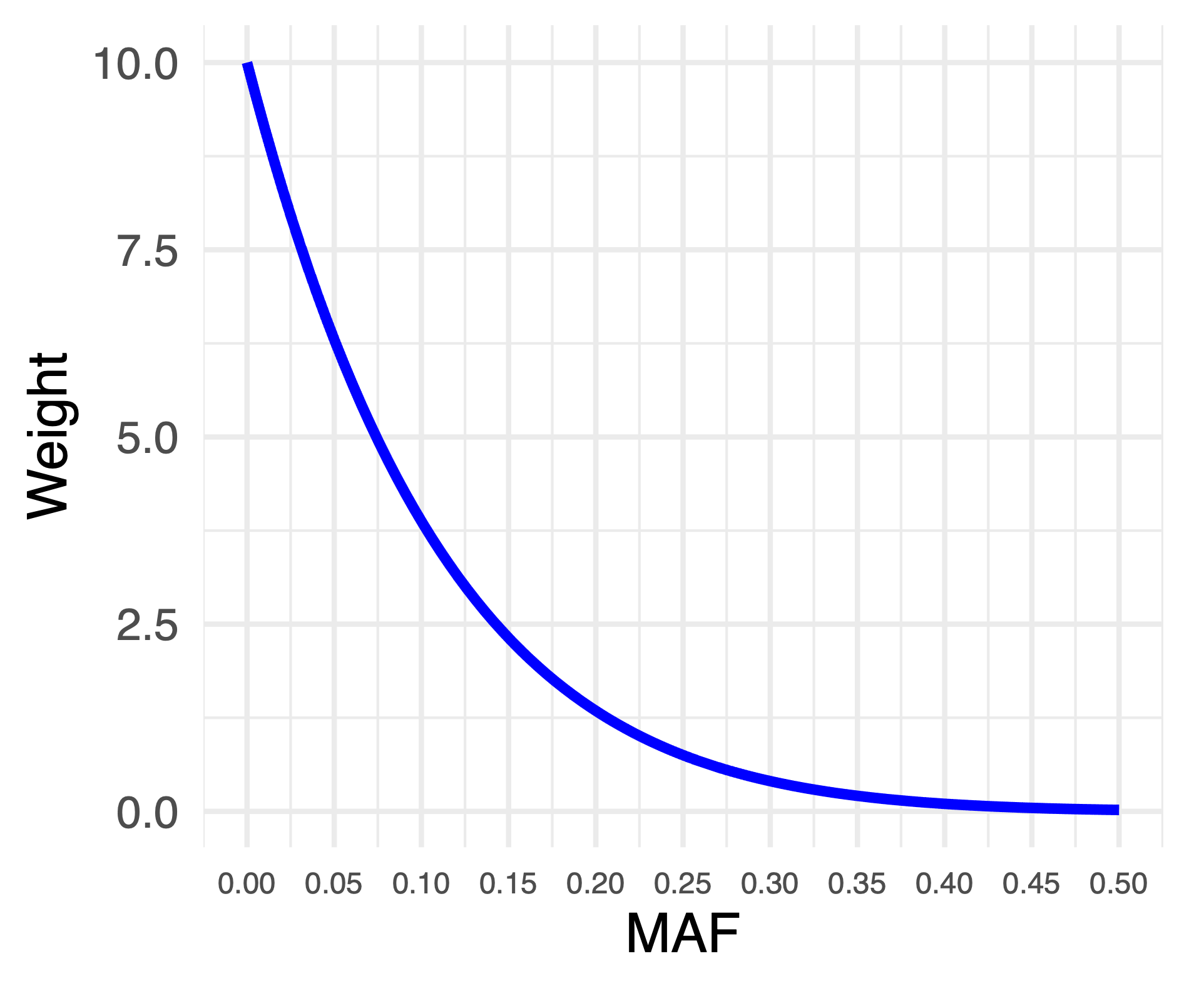


**Supplementary Figure 3. Beta distribution of MAF weights for SKAT-O testing under the dominant model.** The beta distribution curve (beta(MAF, 1, 10)) used to apply weight for variants based on the MAF. The beta value is 10 and the alpha value is 1. Rare variants are assigned higher weights up to 10 compared to the more common variants. MAF, Minor Allele Frequency.

**
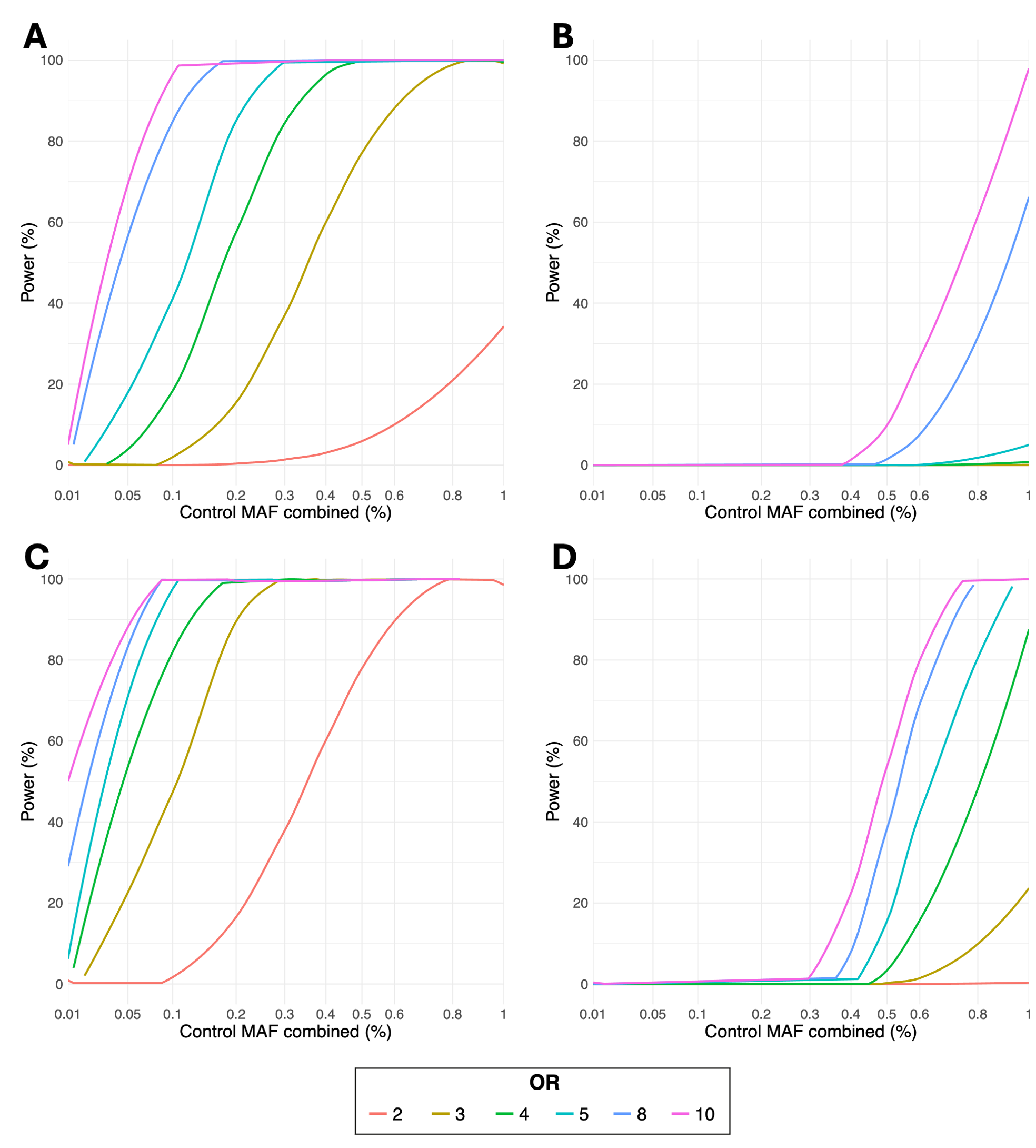
**

**Supplementary Figure 4. Power analysis to identify genes with TGCT-associated variant burden with varying allele frequency and effect .** The simulated power calculations were performed using the current sample size (1,435 cases and 18,284 controls) under (A) a dominant model and (B) a recessive model. Simulated power calculations hypothetical scenario with a larger sample size (10,000 cases and 100,000 controls) were also performed under (C) a dominant model and (D) a recessive model. Similar methodologies as the meta-analysis were used for each model. The target MAF values in controls ranged from 0.01% to 1% and are presented on a square root scale. The target OR values were 2, 3, 4, 5 ,8 and 10. The significance threshold was set at p=6.60x10^-7^ as per the exome-wide analysis.


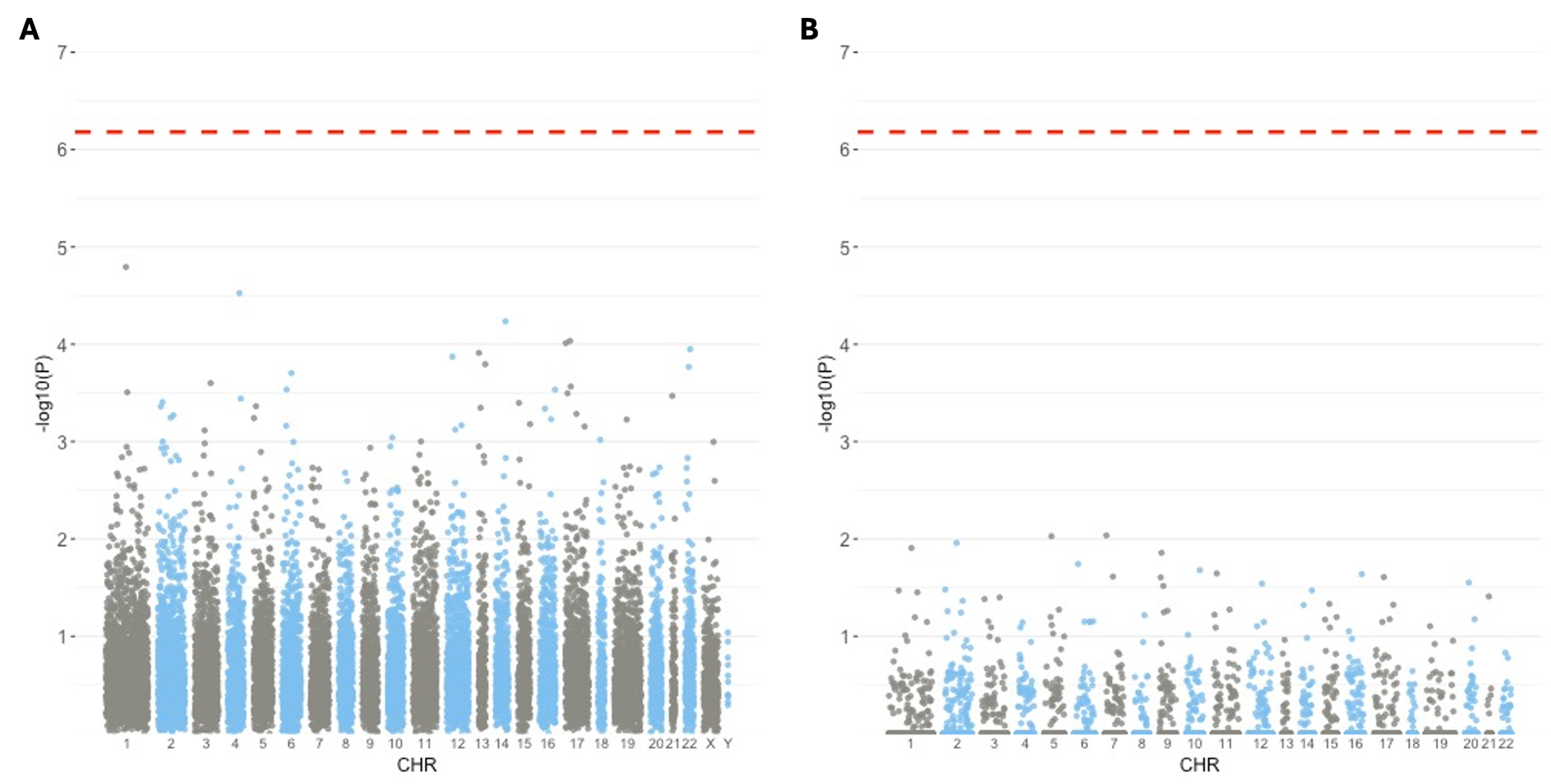


**Supplementary Figure 5. Class II exome-wide gene-level association analysis.** Manhattan plot showing the gene-level statistical results of class II variants exome-wide under a (A) dominant model (≥1 qualifying variants per gene) using SKAT-O testing and including X and Y-linked genes and (B) a recessive model using Fisher’s exact testing (≥2 qualifying variants per gene). The x-axis denotes the genomic positions of genes in every chromosome, while the y-axis represents the negative base-10 logarithm (log10) of the *p*-values. The horizontal red dotted line represents the Bonferroni-corrected significance threshold (p= 6.60x10^-7^ or -log10(*P*)= 6.18, corresponding to 75,760 tests); no gene is significantly associated at this threshold.


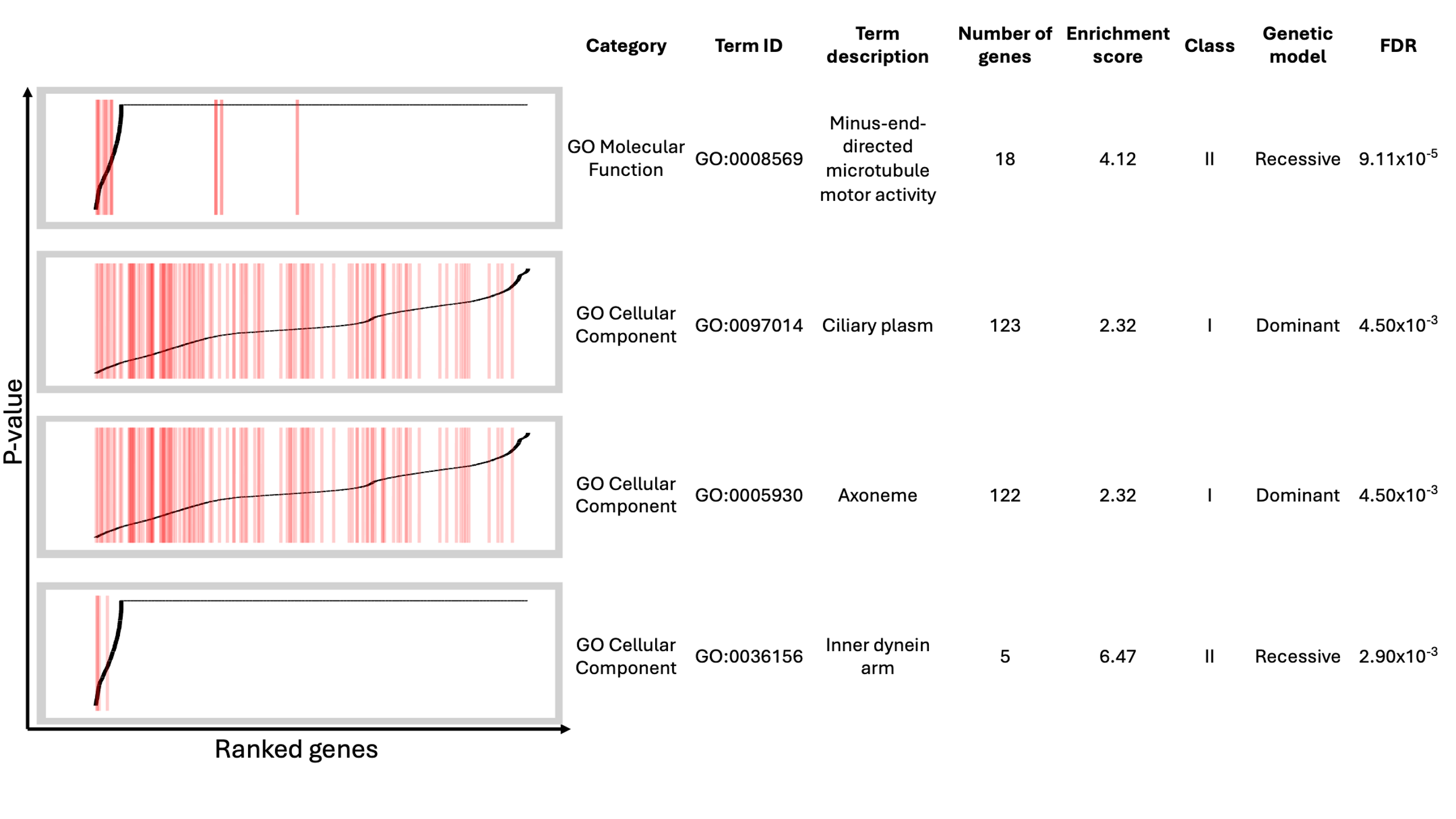


**Supplementary Figure 6. Functional enrichment analysis of GO category gene sets using exome-wide association-derived *p*-values.** Gene-set enrichment analysis of class I and II variants ranked by gene-level *p*-value following a dominant and recessive model. Enrichment was evaluated using the gene ontology (GO) databases within STRING software. The significance threshold was set at FDR <0.01 and enrichment score >2 to limit false positives. Only significantly enriched results among GO categories are presented. *P*-value distributions under a given class and genetic model are presented and the red bands represent the genes from the corresponding significantly enriched GO category. FDR, false discovery rate; GO, gene ontology.

# Supplementary Methods

## Studies, subjects and data for primary analysis

The first set of Testicular Germ Cell Tumour (TGCT) cases comprised a whole exome sequencing (WES) series ascertained from (i) ‘The UK Genetics of Testicular Cancer Study' and (ii) ‘Identification, epidemiological and molecular analyses of families with susceptibility to TGCT' (recruitment via the UK Testicular Cancer Collaboration and International Testicular Cancer Linkage Consortium) which were coordinated by The Institute of Cancer Research (ICR). Out of 986 cases, 335 were reported to be familial cases and 651 are sporadic cases. Collection of blood samples and clinical information from all subjects was undertaken after obtaining informed written consent and relevant ethical review board approval at the respective institutions. Written informed consent was obtained from all individuals with ethical review board approval (UK National Cancer Research Network Multi-Research Ethics Committee—MREC02/06/66, 06/MRE06/41) and the study was conducted in accordance with the declaration of Helsinki.

The second set of TGCT patients were enrolled in The Cancer Genome Atlas (TCGA) from 15 referral centres and 150/379 germline samples were sent for WES, which were used in this study.

From the UK Biobank (UKB), WES data for 658 TGCT germline samples were drawn from the cloud-based UKB Research Analysis Platform (RAP) ([https://ukbiobank.dnanexus.com](https://ukbiobank.dnanexus.com/)) enabled by DNAnexus and powered by Amazon Web Services, using ICD10 codes C62 from linkage to cancer registrations.

The 18,400 UKB controls were randomly selected from a pre-curated subset of samples (>133,000) with self-reported European ancestry and WES data were also extracted for these individuals to maintain a minimum of 10:1 control-case ratio after sample quality control (QC). Further details on the TCGA and UKB datasets can be found in prior publications^1,2^. No initial familial cases were reported/found in TCGA and UKB samples in this analysis.

For ICR and TCGA, histological subtype was as submitted by the referring clinician on referral to the research study; for UK Biobank, the subtype reflects the histology information submitted upon registration of the cancer to the English National Disease Registration Service.

With the three datasets we had access to, the project was strategically carried out in three separate pillars for each TGCT case set (ICR, TCGA and UKB). Different numbers of non-overlapping UKB controls were assigned for each pillar: 10,300 controls for the ICR pillar, 1,500 controls for the TCGA pillar and 6,600 controls for the UKB pillar.

## Studies, subjects and datasets using for quality control analyses

The 1000 Genomes phase 3 data (1000 Genomes Project Consortium et al.^3^) were used as a supporting genetic dataset for the principal component analysis during sample QC as described below. The ICR control series comprised 1,581 cancer-free individuals from the UK 1958 Birth Cohort, 993 of which are from the ICR1000 UK exome series (EGAD00001001021) and 588 from the ICR MOPOPDGEN UK exome control series (EGAD00001004396). These controls were used for intermediary variant QC but not for the main association tests.

## Sample preparation and sequencing

Samples from ICR cases were processed as per the following: One ug of DNA from germline blood samples from each individual was fragmented using a Covaris E Series instrument (Covaris Inc. Woburn, MA, USA). Preparation of indexed paired-end libraries was done using Illumina TruSeq 62 Mb expanded exome enrichment kit (Illumina, San Diego, CA, USA). Sequencing was conducted using Illumina HiSeq2000 or 2500 technology, generating paired-end reads with a length of 100 base pairs each (2 × 100 bp). Truncated or malformed FASTQ files were excluded (*N* = 1 sample). CASAVA (v1.8) was used to generate per-read FASTQ files from per-cycle BCL basecall files. For the ICR controls, paired-end FASTQ files were generated for an initial total of *N* = 1,648 samples. Truncated or malformed FASTQ files were excluded (*N* = 67 samples). TCGA and UK Biobank samples were prepared and sequenced as previously described in Supplementary Table 3 and previous publications^1^. In brief, TCGA exomes were captured using the HGSC VCRome 2.1 design (42Mb, NimbleGen) and sequenced with the Illumina HiSeq2000 technology. The UKB samples were prepared with the IDT xGen Exome Research Panel v1.0 (39Mb) and sequenced with the Illumina NovaSeq 6000 platform.

## Sequencing alignment, variant calling and joint genotyping pipeline

FASTQ files for ICR and TCGA samples were pre-processed using the nf-core/sarek pipeline (v.2.7) using NextFlow (v.20.10.0). The pipeline and all related software dependencies were implemented via a Singularity image (converted from a docker image using Singularity (v.3.6.1)). Runs were monitored using NextFlow Tower (v.21.12.0). The pipeline was used to first trim adaptor sequences using Trim Galore (version 0.6.5). The reads were then mapped to reference build GRCh38 using bwa-mem2 (v.2.0) in CRAM format and duplicates were marked using GATK4 MarkDuplicates^4^. The pipeline computed and applied a base quality score recalibration (BQSR). QC metrics including sequence quality, sequence content, per-base N content and sequence length were generated using multiQC (v.1.8). These metrics were assessed before and after trimming of sequence files. In the ICR and TCGA datasets, upon observing primary reads with split alignments (SA) to unlocalized contig decoys and enriched in variants relative to reads with no SA flags, they were filtered out to mitigate the risk of false positive variant calling from misalignment. The median proportion of removed reads was 0.02% and 0.05% for the ICR and the TCGA case sets, respectively. The UKB samples were processed using the OQFE protocol as described in a previous publication^2^. Variant calling was performed using Google’s DeepVariant (v.1.4.0) to produce single-sample genomic variant call format (gVCF) files for both ICR and TCGA cases^5^. Joint genotyping was performed on the resulting gVCFs along with those of UK Biobank using GLNexus (v1.0.4) to produce Project VCF (pVCF)^5^. A Browser Extensible Data (BED) file was used as an additional input to target the protein-coding regions while also including 20bp into the introns, which contain canonical splice sites, and the 5’ untranslated regions (5’UTR) implicated in transcriptional regulation. The joint genotyping was conducted on the UKB RAP ([https://ukbiobank.dnanexus.com](https://ukbiobank.dnanexus.com/)).

## Sample level quality control

### Pre-variant calling quality control

The 30X coverage and error rate were used as pre-variant calling sequencing metrics to remove outliers from further analyses. Outliers were identified on the basis of 4 more median absolute deviations (MADs) in both directions from the median for both metrics. This step was applied to the ICR and TCGA datasets only.

### Post-variant calling quality control

The following post-variant calling sequencing metrics were produced using Picardtools (v2.27.1): number of SNPs, number of INDELs, SNP:INDEL ratio, transition-to-transversion (Ti:Tv) ratio. The 4 x MADs in both directions were also used to remove outliers. This step was conducted on the UKB RAP.

### Genomically inferred sex

We assessed heterozygosity of the X chromosome to identify disparities in reported sex. The homologous sequence regions defined as pseudoautosomal regions were pruned out using PLINK^6^. The X inbreeding coefficient was estimated and a minimum threshold of 0.4 was set to exclude samples with no genetic confirmation of male sex. This step was conducted on the UKB RAP.

### Established and cryptic relatedness

We checked for established and cryptic relatedness (up to 3-degree relationships) amongst the case and control series by inferring kinship using the KING toolset (v2.2.9). Samples with high relatedness (KING kinship value >0.0442), up to and including 3^rd^ degree relatives are eliminated^7^.

### Ancestry exclusions

We performed genetic ancestry analysis using Principal Component Analysis (PCA). We used the 1000 Genomes phase 3 data as a reference to aid in the identification of subpopulations. PCA was performed using PLINK using a set of shared autosomal SNPs of missingness <5% and a minor allele frequency (MAF) >1%. The first two principal components were computed using non-Finnish European samples within the 1000 Genomes dataset as the target reference population and a scaling factor of 1.6. All case samples for whom Euclidean distance from the centre fell outside the radius specified by the maximum Euclidean distance of the 1000 Genomes non-Finnish European data, multiplied by the scaling factor, were considered non-European and excluded from the study. This resulted in the exclusion of 19 case samples from the analysis across ICR, TCGA and UKB (Supplementary Table 4).

## Variant level quality control

### Variant normalisation

After sample exclusions, multiple QC steps at the variant level followed. The first steps included variant normalisation, which ensured variants are parsimonious and left aligned for consistent representation, description and nomenclature of variants^8^. To make variants parsimonious, they will be represented in as few nucleotides as possible without an allele length of 0. The left alignment is performed by shifting the starting position of variants to the left until it is no longer possible to do so. Then an allele count AC>0 filter was applied to exclude variants that were only called in other datasets before their separation into three pillars.

### Variant set calibration and selection

We next implemented a variant set calibration process with varying stringency levels across 4 parameters^9,10^: (i) binomial test significance threshold during coverage normalisation (from 1.00E-05 to 1.00E-08), (ii) Hardy-Weinberg Equilibrium (HWE) significance threshold (from 1.00E-07 to 1.00E-09), (iii) Coverage normalisation depth cut-off (10X, 8X and 5X) and (iv) low complexity (LC) filter degree of stringency (defined as loose and stringent, as per the GIAB). The combination of the varying content for each parameter produced 72 combinations in total for each pillar (Supplementary Table 6). The 95^th^ percentile lambda value used as genomic control ranged from 1.14 to 1.23 for the ICR and TCGA datasets, showing inflation overall but minimal differences between variant sets. The lambda value for UKB dataset showed an optimal profile with lambda values close to 1. Thus, the heterogeneity between ICR/TCGA cases and UKB controls likely explains the observed inflation. Nonetheless, the selected variant sets for every pillar used the loosest criteria for 3/4 parameters, with the exception of the low complexity parameter, which was set at the strict level as defined by the GIAB to avoid the impact of large tandem repeats >200bp. Loosening 3/4 parameters allows for the retention of higher number of variants while keeping the Ti:Tv ratio above 2.8 across all pillars to reduce false positives^11^. Coverage normalisation was performed to exclude sites with different coverage between cases and controls. Two alternative coverage criteria were then applied: (i) variants with ≥5X coverage in 75% of samples tested were retained, (ii) otherwise, variants could be retained at sites covered ≥5X in >10% of samples if passing a binomial test (p <1 × 10^-8^) applied to evaluate differences in proportions between cases and controls. Variants violating HWE (p <1 × 10^-9^) were eliminated using PLINK2^12^. Low complexity regions as described by the GIAB (≥7bp perfect homopolymers, ≥10bp imperfect homopolymers, ≥11bp dinucleotide tandem repeats, ≥15bp trinucleotide tandem repeats, $\geq$20bp quadnucleotide tandem repeats) were excluded. A mappability check as per the GIAB across all 72 variant sets was performed within a 100bp window (fewer than 2 mismatched SNPs and fewer than 1 indel <15bp). We retained variants with at least one high quality (HQ) heterozygous genotype across cases and controls. Heterozygous genotypes passed the HQ filter if they met the following criteria: depth (DP) ≥10, genotype quality (GQ) ≥20 and allelic depth ratio >0.2.

### ICR control QC implementation

For the ICR pillar an additional comparison between the UKB controls and 1,581 in-house healthy controls was performed to limit false positives. The ICR controls were joint-genotyped with the ICR cases and UKB controls. We then intersected with the post-QC variant set from the main pillar to keep the sites of interest. Afterwards, a two-sided binomial test was applied to exclude variants for which genotype proportions differed markedly between control sets (p <1 × 10^-5^).

### Additional variant QC to remove observed false positives

Following observation of artifacts leading to spurious association signals, we conducted additional QC steps to optimize specificity and eliminate false positives. We implemented an additional mappability filter based on the University of California, Santa Cruz (UCSC) browser tracks (alignability in 100bp window = 1) to minimize false positive variants. Regions with proportion expressed across transcripts (pext)^13^ scores below 10% in testis were excluded (Supplementary Fig. 1). The pext values were downloaded from gnomAD (v2.1.1), lifted over from build 37 to 38 using the UCSC genome browser and formatted as a BED file. Following the sex mismatch filter during sample-level QC, we also set as missing the filter for discordant heterozygous genotypes on the X and Y chromosomes to reduce false positive associations in sex chromosome genes from low confidence genotype calls. The median genotype quality (GQ) per variant was calculated separately for cases and controls for each group of genotypes (homozygous wild-type, heterozygous and homozygous alternate). A threshold of median GQ>10 was applied to filter out variants with an overwhelming proportion of low confidence genotype calls across samples.

## Variant annotation and stratification

Annotations were generated for all transcripts using VEP cache version 110. Multiple annotations and VEP plugins were included:

- Gene symbol and name
- Variant impact and consequence
- Human Genome Variation Society (HGVS) nomenclature
- Loss-Of-Function Transcript Effect Estimator (LOFTEE, v.1.0.4; Karczewski et al.^14^).
- Combined Annotation Dependent Depletion (CADD) (v1.6) scores (Raw and PHRED-like scaled)^15^
- ClinVar database (2023/06/26 release)

The CSG analysis consisted of one class which combined pathogenic/likely pathogenic ClinVar variants (≥1 star review status) and high-confidence loss of function variants (HC LoF as predicted by LOFTEE). For the five candidate gene sets and the subsequent exome-wide analysis, we grouped variants into two classes: class I: HC LoF and class II: HC LoF plus nonsynonymous variants and inframe indels with a CADD-PHRED score ≥20. As defined by Pyle et al^16^, the GWAS gene set is composed of genes within linkage proximity r^2^ > 0.4 of the most significant candidate SNPs identified by Pluta et al^17^.

## Statistical analyses

The per-base and per-gene depth of coverage was computed using the GATK depthofcoverage tool (v.4.9.1.0). Gene identification was possible with NCBI and MANE annotations in a reference sequence file provided by the UCSC genome table browser. The per-gene proportion of sites above 5X depth for every case sample from every pillar was computed. The median proportion of well-covered sites across each dataset was also subsequently calculated.

Gene-level association testing under a dominant allelic model was implemented in SAIGE-GENE+^18^. A sparse genetic relationship matrix (GRM) was computed using step0 of SAIGE-GENE+ with identical parameters as the corresponding publication (--numRandomMarkerforSparseKin=2000 and --relatednessCutoff=0.125)^18^. Phenotype data was exported as a flat file with the first three principal components as covariates. Urological risk factors (past medical history of cryptorchidism or infertility) and demographic parameters such as smoking, body mass index and alcohol use were not included as covariates due to the inconsistent availability of this information across samples from all datasets. The phenotype data was combined with the sparse GRM to fit a null model using step1 of SAIGE-GENE+. Genotype tests were performed using the pVCF files and the null model using step2 of SAIGE-GENE+. Gene-based analysis under the dominant model, including X and Y-linked genes, was performed using the unified optimal sequence kernel association test (SKAT-O)^19^. A SKAT-O test is a hybrid approach that combines aspects of both burden and SKAT methods while introducing optimal weights for variants. In situations with a mix of variants with similar and varying effects, the more robust SKAT-O test has the ability to adapt and strike a balance between the simplicity of a burden test and the flexibility of a SKAT test. No MAF threshold was set under a dominant model: a MAF weighting, calculated as **beta (MAF, 1, 10),** was instead applied to give higher weights to rare variants while also allowing assessment of the contribution of more common variation (Supplementary Fig. 3). The beta value was brought down from the default value of 25 to a value of 10 to reduce the weight difference between rare and more common variants. This decision was made based on the conclusions of prior studies highlighting the unique underlying polygenic aetiology of TGCT and the substantial contribution of more common variation to the heritability of the disease^20^. A previous publication has also suggested a maximum MAF weight of 10 for rare variants^21^. Lambda GC values were computed from synonymous variants grouped by gene. Under a recessive model, a MAF threshold of 1% was imposed and samples were assigned as carriers for each gene if at least 2 qualifying variants were detected, including assumed compound heterozygosity. This was followed by a Fisher’s exact test comparing frequency of recessive carriers in cases versus controls without the use of any covariates. Using Stouffer’s method, individual *p*-values were converted to weighted Z-scores before calculating the combined *p*-value using the cumulative distribution function of the standard normal distribution. If no qualifying variants were present in a pillar, a flat *p*-value of 1 was assigned to that pillar to allow for the *p*-value combination. Z-score weights were applied from case sample size and gene coverage (proportion of sites above 5X depth) when combining *p*-values between pillars. Correction for multiple testing was performed using a Bonferroni correction. The cumulative significance cut-off for the CSG (136 tests), the five candidate gene set (2,810 tests) and the exome-wide (75,760 tests) analyses were p=3.68x10^-4^, p=1.78x10^-5^ and p=6.60x10^-7^ respectively.

## Power analysis

We generated simulated VCF files, genetic relationship matrices (GRMs), and associated phenotype files for a binary trait using Python 3. The same case and control sample sizes as the current study following sample QC were used (1,435 cases and 18,284 controls). We ran the simulation with 10,000 samplings for every possible combination of MAF_combined_ in control samples (at levels of 0.0001, 0.0002, 0.0005, 0.001, 0.002, 0.005, 0.006, 0.008 and 0.01) and genes with qualifying variants of varying effect sizes (odds ratios of 2, 3, 4, 5, 8, and 10). The simulation was run under a dominant and a recessive model akin to the methods employed in the main analysis. Under the dominant model, we executed the SAIGE-GENE+ workflow using the input files and the combination of the parameters mentioned above. Upon obtaining the SKAT-O results under the dominant model and the Fisher’s exact results under the recessive model, we calculated the proportion of genes with a *p*-value that crosses a significance threshold of p=6.60x10^-7^. Those proportions were characterized as the statistical power. The power analysis was rerun with a hypothetical future scenario containing a larger sample size (10,000 cases and 100,000 controls) to assess the potential gain in power compared to our study.

## Functional enrichment analysis

Using the STRING software, we sought to evaluate enrichment of exome-wide association signals across functional gene groupings in the three gene ontology (GO) categories (biological process, cellular component and molecular function) and then expanded to incorporate other databases, including InterPro, Pfam, Reactome and the built-in STRING clusters. We then investigated pathways related to cytoskeletal structure and function based on previous evidence of cilia-microtubule genes, including the dynein axonemal assembly factor 1 (*DNAAF1*) gene, being implicated in familial TGCT^22^.

Characterization of functional categories as microtubule-, ciliary- or dynein-related was conducted using a keyword search (with keywords "microtubule", "axoneme", "axonemal", "dynein", "cilia", "ciliary", "cytoskeleton") across the names of all groupings within the GO dataset and expanded databases. We assessed excess enrichment in microtubule/cilia functional groupings as a whole relative to all other gene sets with a Fisher’s exact test.

## References (supplementary methods)

1. Shen H, Shih J, Hollern DP, et al. Integrated Molecular Characterization of Testicular Germ Cell Tumors. *Cell Rep*. Jun 12 2018;23(11):3392-3406. doi:10.1016/j.celrep.2018.05.039

2. Szustakowski JD, Balasubramanian S, Kvikstad E, et al. Advancing human genetics research and drug discovery through exome sequencing of the UK Biobank. *Nat Genet*. Jul 2021;53(7):942-948. doi:10.1038/s41588-021-00885-0

3. Genomes Project C, Auton A, Brooks LD, et al. A global reference for human genetic variation. *Nature*. Oct 1 2015;526(7571):68-74. doi:10.1038/nature15393

4. Bathke J, Luhken G. OVarFlow: a resource optimized GATK 4 based Open source Variant calling workFlow. *BMC Bioinformatics*. Aug 13 2021;22(1):402. doi:10.1186/s12859-021-04317-y

5. Yun T, Li H, Chang PC, Lin MF, Carroll A, McLean CY. Accurate, scalable cohort variant calls using DeepVariant and GLnexus. *Bioinformatics*. Apr 5 2021;36(24):5582-5589. doi:10.1093/bioinformatics/btaa1081

6. Purcell S, Neale B, Todd-Brown K, et al. PLINK: a tool set for whole-genome association and population-based linkage analyses. *Am J Hum Genet*. Sep 2007;81(3):559-75. doi:10.1086/519795

7. Manichaikul A, Mychaleckyj JC, Rich SS, Daly K, Sale M, Chen WM. Robust relationship inference in genome-wide association studies. *Bioinformatics*. Nov 15 2010;26(22):2867-73. doi:10.1093/bioinformatics/btq559

8. Tan A, Abecasis GR, Kang HM. Unified representation of genetic variants. *Bioinformatics*. Jul 1 2015;31(13):2202-4. doi:10.1093/bioinformatics/btv112

9. Loveday C, Garrett A, Law P, et al. Analysis of rare disruptive germline mutations in 2135 enriched BRCA-negative breast cancers excludes additional high-impact susceptibility genes. *Ann Oncol*. Dec 2022;33(12):1318-1327. doi:10.1016/j.annonc.2022.09.152

10. Guo MH, Plummer L, Chan YM, Hirschhorn JN, Lippincott MF. Burden Testing of Rare Variants Identified through Exome Sequencing via Publicly Available Control Data. *Am J Hum Genet*. Oct 4 2018;103(4):522-534. doi:10.1016/j.ajhg.2018.08.016

11. Carson AR, Smith EN, Matsui H, et al. Effective filtering strategies to improve data quality from population-based whole exome sequencing studies. *BMC Bioinformatics*. May 2 2014;15:125. doi:10.1186/1471-2105-15-125

12. Chang CC, Chow CC, Tellier LC, Vattikuti S, Purcell SM, Lee JJ. Second-generation PLINK: rising to the challenge of larger and richer datasets. *Gigascience*. 2015;4:7. doi:10.1186/s13742-015-0047-8

13. Cummings BB, Karczewski KJ, Kosmicki JA, et al. Transcript expression-aware annotation improves rare variant interpretation. *Nature*. May 2020;581(7809):452-458. doi:10.1038/s41586-020-2329-2

14. Karczewski KJ, Francioli LC, Tiao G, et al. The mutational constraint spectrum quantified from variation in 141,456 humans. *Nature*. May 2020;581(7809):434-443. doi:10.1038/s41586-020-2308-7

15. Rentzsch P, Schubach M, Shendure J, Kircher M. CADD-Splice-improving genome-wide variant effect prediction using deep learning-derived splice scores. *Genome Med*. Feb 22 2021;13(1):31. doi:10.1186/s13073-021-00835-9

16. Pyle LC, Kim J, Bradfield J, et al. Germline Exome Sequencing for Men with Testicular Germ Cell Tumor Reveals Coding Defects in Chromosomal Segregation and Protein-targeting Genes. *Eur Urol*. May 26 2023;doi:10.1016/j.eururo.2023.05.008

17. Pluta J, Pyle LC, Nead KT, et al. Identification of 22 susceptibility loci associated with testicular germ cell tumors. *Nat Commun*. Jul 23 2021;12(1):4487. doi:10.1038/s41467-021-24334-y

18. Zhou W, Bi W, Zhao Z, et al. SAIGE-GENE+ improves the efficiency and accuracy of set-based rare variant association tests. *Nat Genet*. Oct 2022;54(10):1466-1469. doi:10.1038/s41588-022-01178-w

19. Lee S, Emond MJ, Bamshad MJ, et al. Optimal unified approach for rare-variant association testing with application to small-sample case-control whole-exome sequencing studies. *Am J Hum Genet*. Aug 10 2012;91(2):224-37. doi:10.1016/j.ajhg.2012.06.007

20. Loveday C, Law P, Litchfield K, et al. Large-scale Analysis Demonstrates Familial Testicular Cancer to have Polygenic Aetiology. *Eur Urol*. Sep 2018;74(3):248-252. doi:10.1016/j.eururo.2018.05.036

21. Curtis D. Exploration of weighting schemes based on allele frequency and annotation for weighted burden association analysis of complex phenotypes. *Gene*. Jan 30 2022;809:146039. doi:10.1016/j.gene.2021.146039

22. Litchfield K, Levy M, Dudakia D, et al. Rare disruptive mutations in ciliary function genes contribute to testicular cancer susceptibility. *Nat Commun*. Dec 20 2016;7:13840. doi:10.1038/ncomms13840
